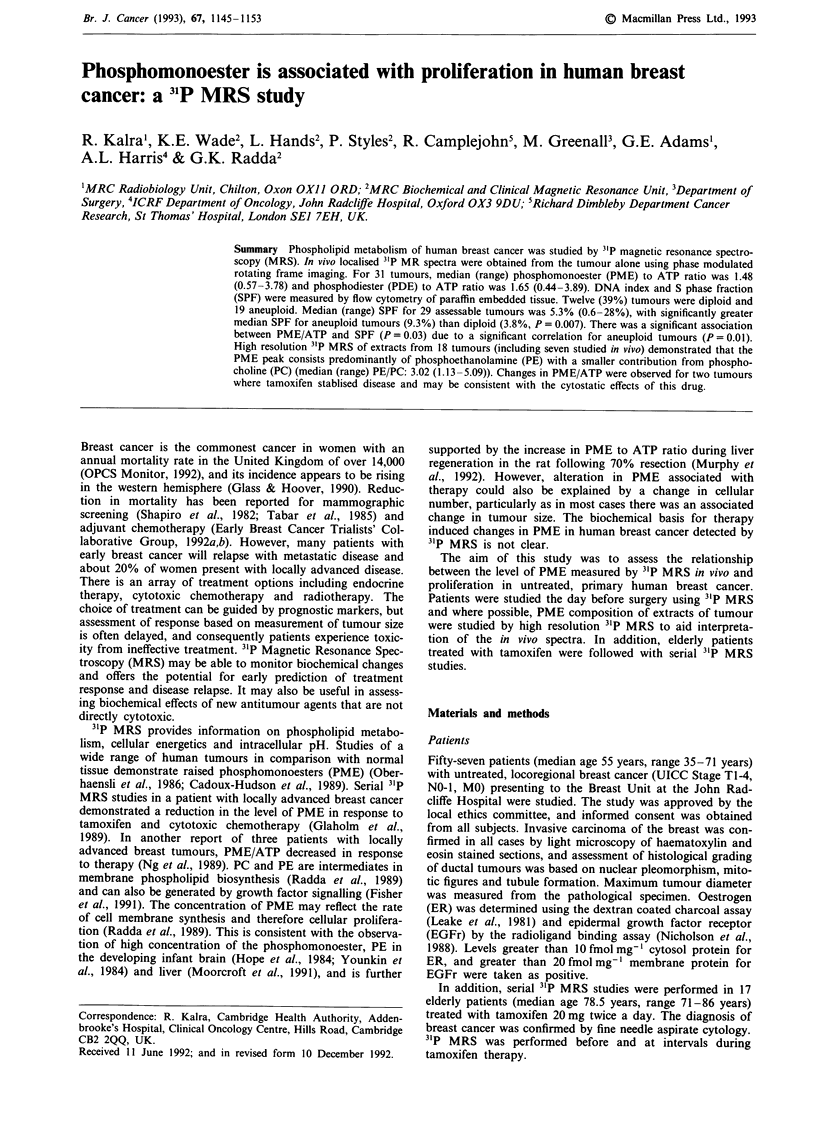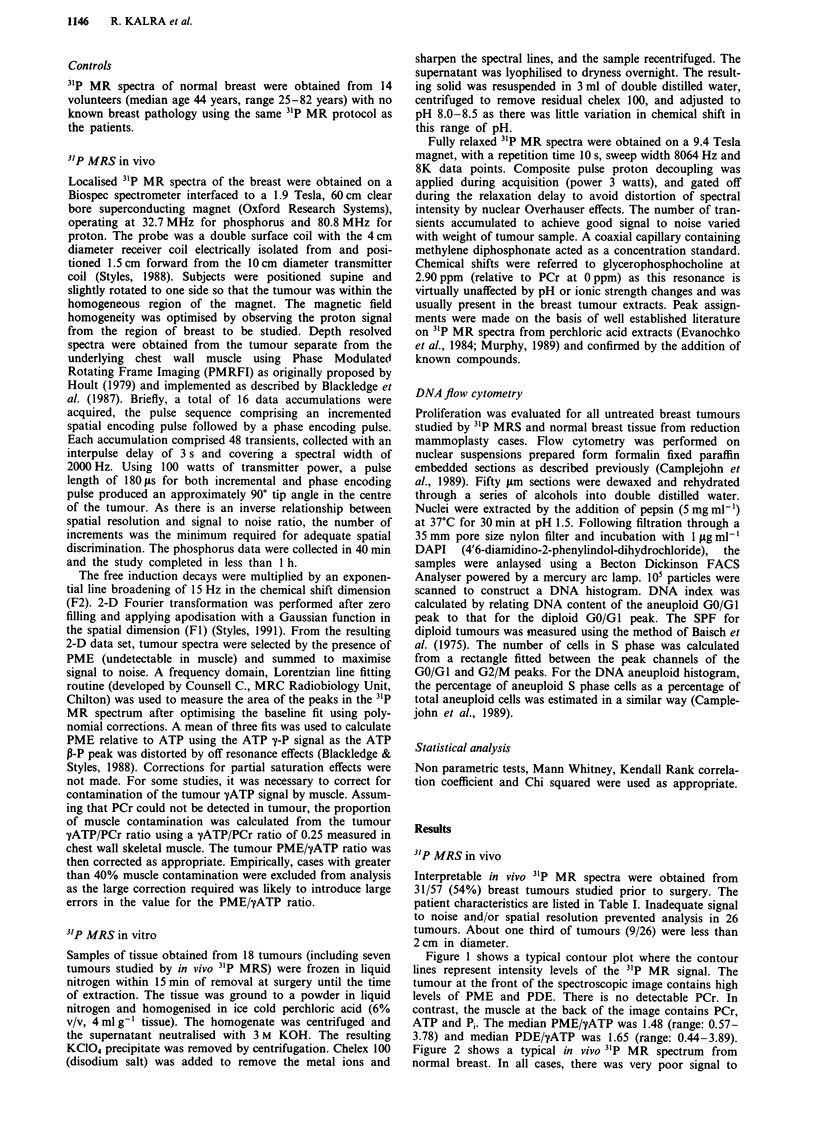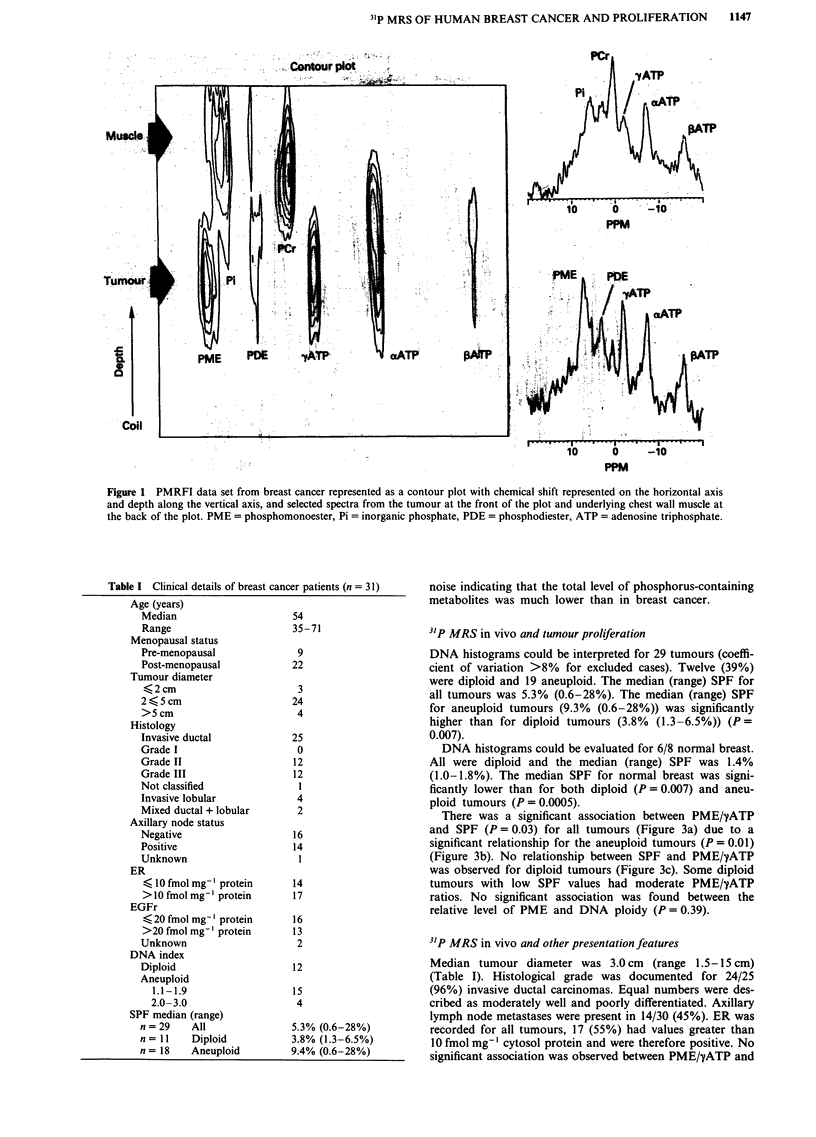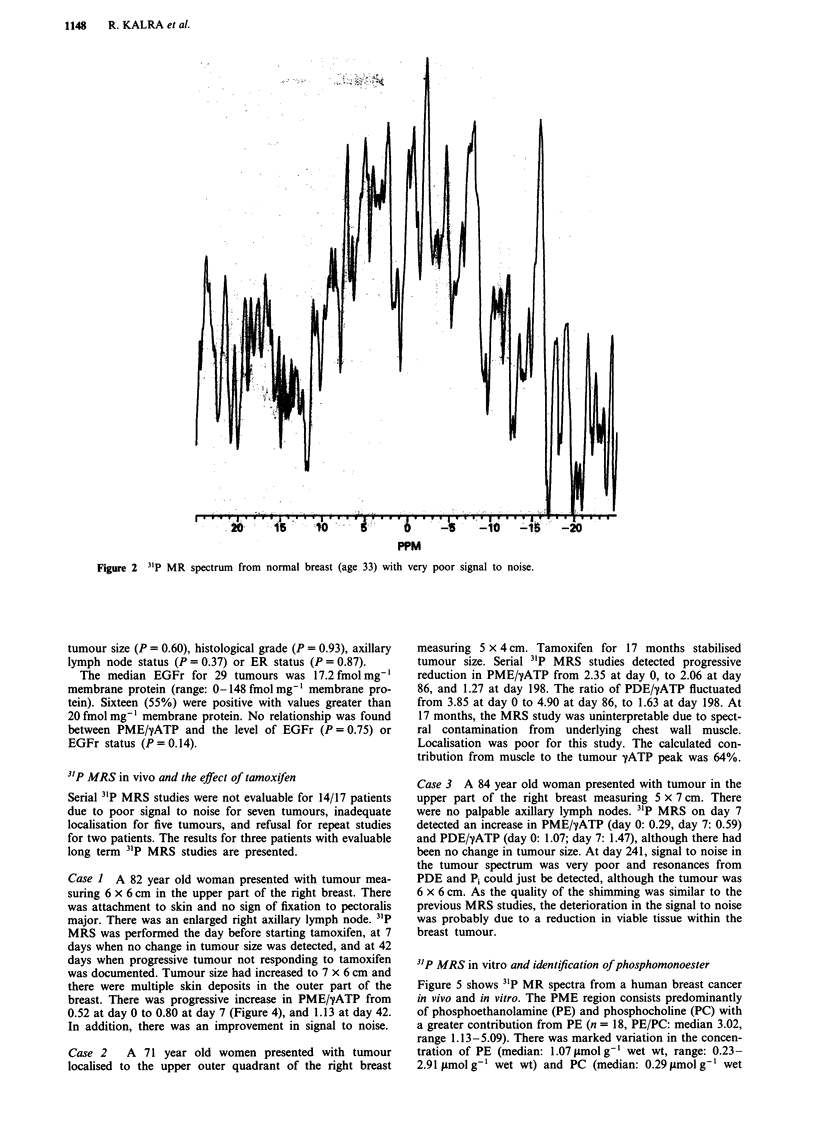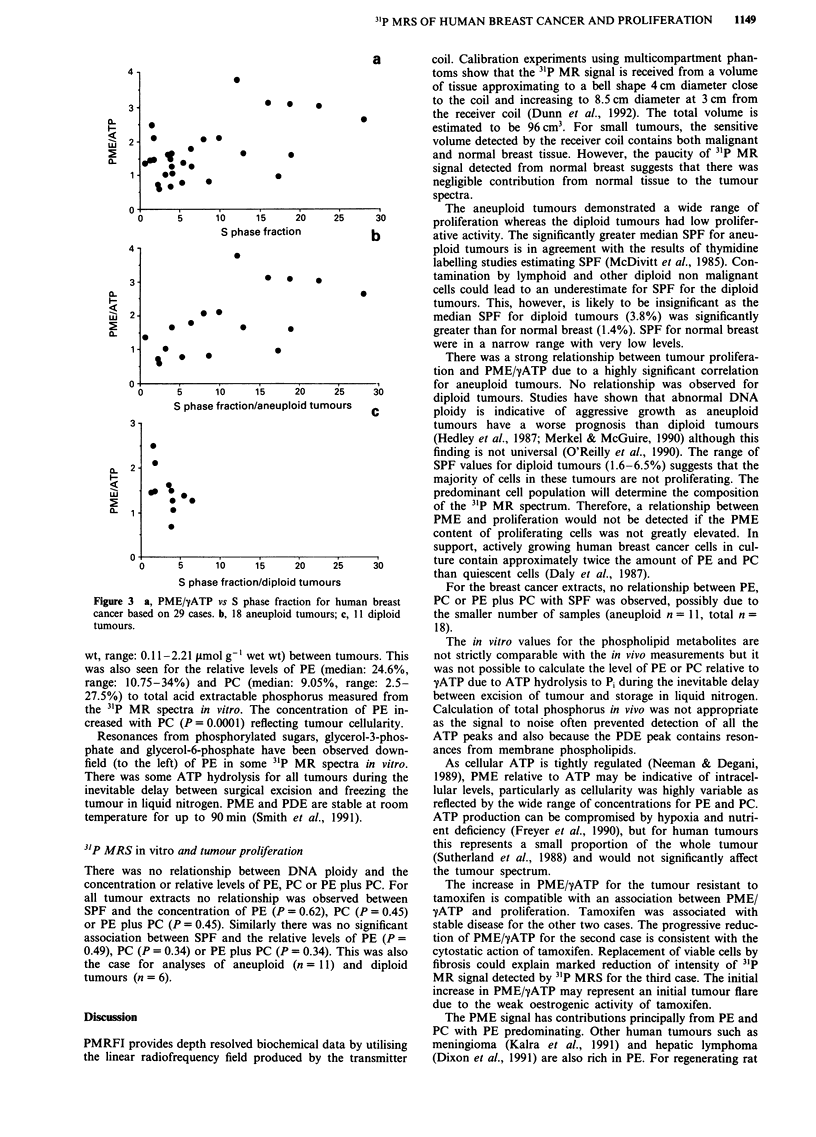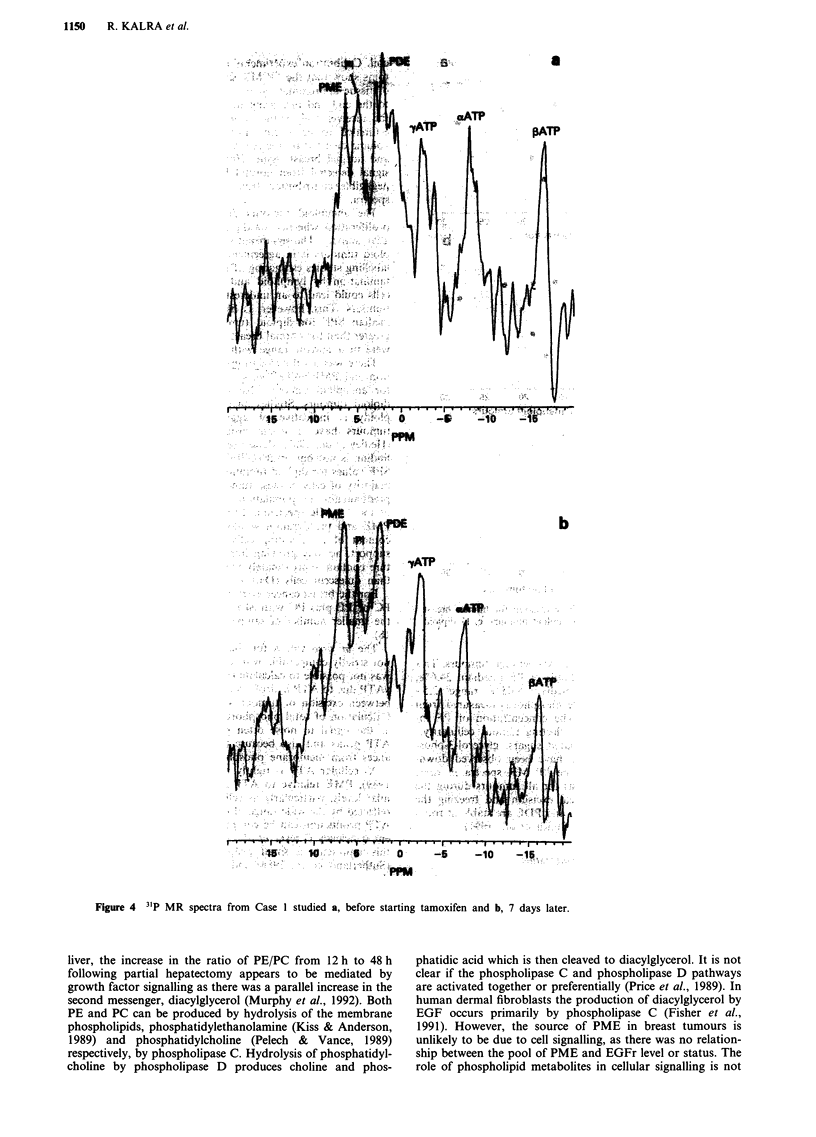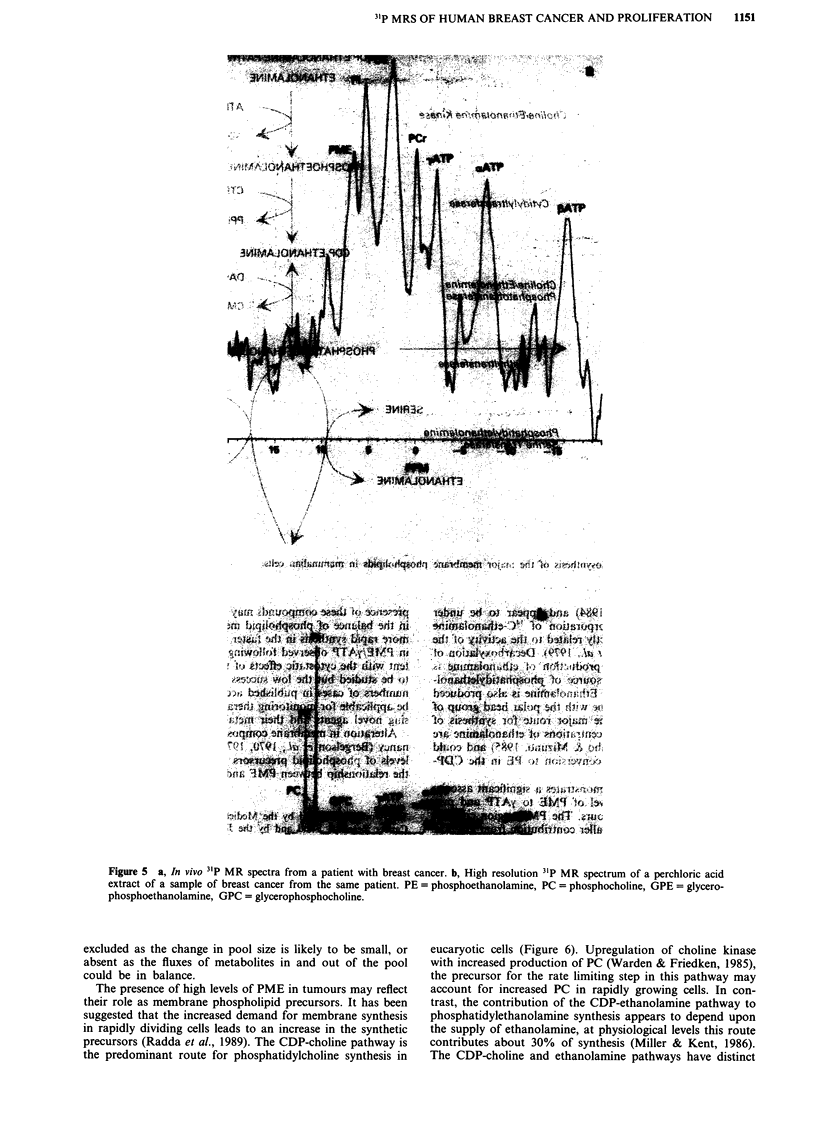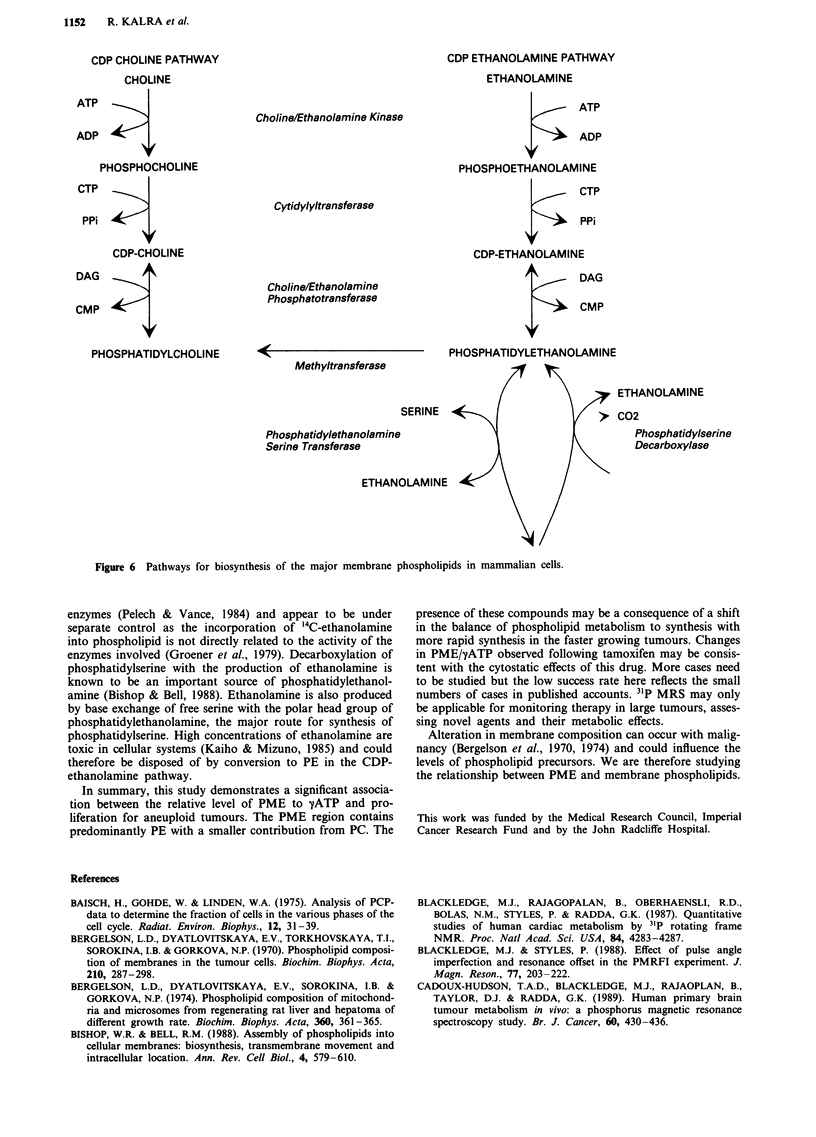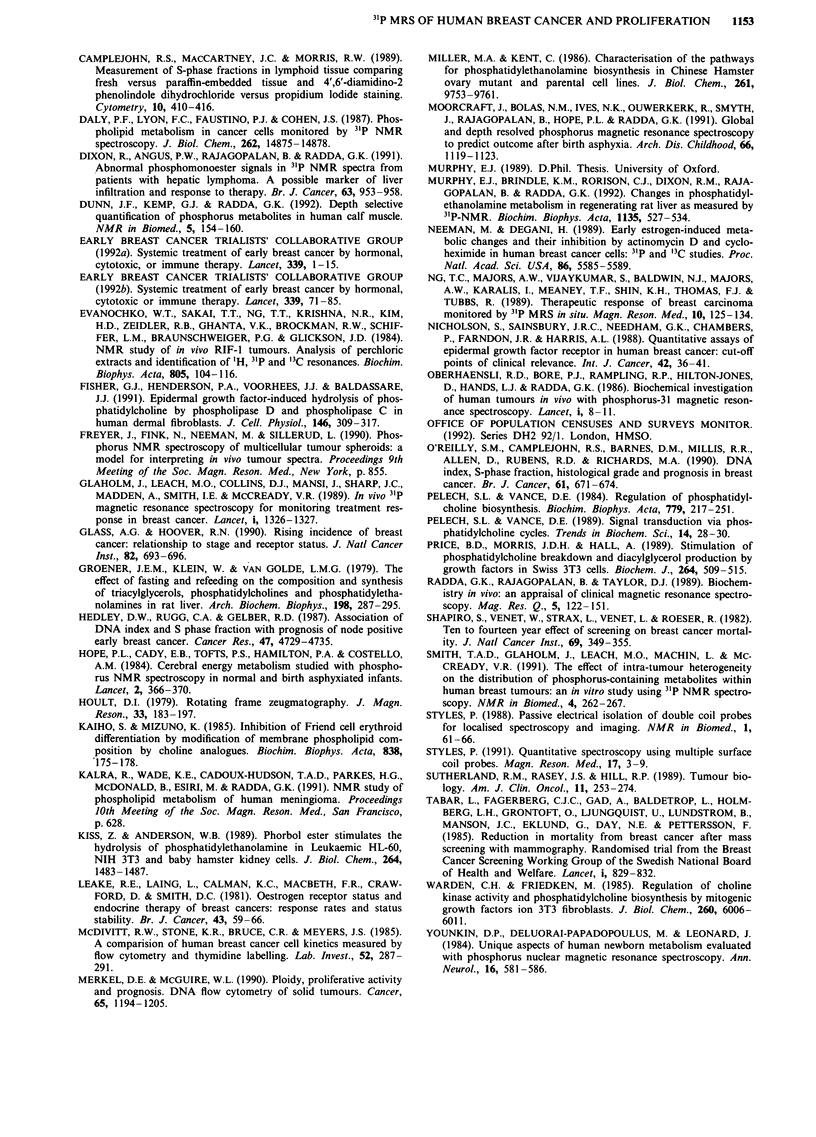# Phosphomonoester is associated with proliferation in human breast cancer: a 31P MRS study.

**DOI:** 10.1038/bjc.1993.211

**Published:** 1993-05

**Authors:** R. Kalra, K. E. Wade, L. Hands, P. Styles, R. Camplejohn, M. Greenall, G. E. Adams, A. L. Harris, G. K. Radda

**Affiliations:** MRC Radiobiology Unit, Chilton, Oxon, UK.

## Abstract

Phospholipid metabolism of human breast cancer was studied by 31P magnetic resonance spectroscopy (MRS). In vivo localised 31P MR spectra were obtained from the tumour alone using phase modulated rotating frame imaging. For 31 tumours, median (range) phosphomonoester (PME) to ATP ratio was 1.48 (0.57-3.78) and phosphodiester (PDE) to ATP ratio was 1.65 (0.44-3.89). DNA index and S phase fraction (SPF) were measured by flow cytometry of paraffin embedded tissue. Twelve (39%) tumours were diploid and 19 aneuploid. Median (range) SPF for 29 assessable tumours was 5.3% (0.6-28%), with significantly greater median SPF for aneuploid tumours (9.3%) than diploid (3.8%, P = 0.007). There was a significant association between PME/ATP and SPF (P = 0.03) due to a significant correlation for aneuploid tumours (P = 0.01). High resolution 31P MRS of extracts from 18 tumours (including seven studied in vivo) demonstrated that the PME peak consists predominantly of phosphoethanolamine (PE) with a smaller contribution from phosphocholine (PC) (median (range) PE/PC: 3.02 (1.13-5.09)). Changes in PME/ATP were observed for two tumours where tamoxifen stablized disease and may be consistent with the cytostatic effects of this drug.


Br. J. Cancer (1993), 67, 1145–1153 © Macmillan Press Ltd., 1993

### Phosphomonoester is associated with proliferation in human breast cancer: a $^{31}$P MRS study

R. Kalra[1], K.E. Wade[2], L. Hands[2], P. Styles[2], R. Camplejohn[5], M. Greenall[3], G.E. Adams[1], A.L. Harris[4] & G.K. Radda[2]

[1]*MRC Radiobiology Unit, Chilton, Oxon OX11 ORD;* [2]*MRC Biochemical and Clinical Magnetic Resonance Unit,* [3]*Department of Surgery,* [4]*ICRF Department of Oncology, John Radcliffe Hospital, Oxford OX3 9DU;* [5]*Richard Dimbleby Department Cancer Research, St Thomas' Hospital, London SE1 7EH, UK.*

**Summary** Phospholipid metabolism of human breast cancer was studied by $^{31}$P magnetic resonance spectroscopy (MRS). *In vivo* localised $^{31}$P MR spectra were obtained from the tumour alone using phase modulated rotating frame imaging. For 31 tumours, median (range) phosphomonoester (PME) to ATP ratio was 1.48 (0.57–3.78) and phosphodiester (PDE) to ATP ratio was 1.65 (0.44–3.89). DNA index and S phase fraction (SPF) were measured by flow cytometry of paraffin embedded tissue. Twelve (39%) tumours were diploid and 19 aneuploid. Median (range) SPF for 29 assessable tumours was 5.3% (0.6–28%), with significantly greater median SPF for aneuploid tumours (9.3%) than diploid (3.8%, $P = 0.007$). There was a significant association between PME/ATP and SPF ($P = 0.03$) due to a significant correlation for aneuploid tumours ($P = 0.01$). High resolution $^{31}$P MRS of extracts from 18 tumours (including seven studied *in vivo*) demonstrated that the PME peak consists predominantly of phosphoethanolamine (PE) with a smaller contribution from phosphocholine (PC) (median (range) PE/PC: 3.02 (1.13–5.09)). Changes in PME/ATP were observed for two tumours where tamoxifen stablised disease and may be consistent with the cytostatic effects of this drug.

Breast cancer is the commonest cancer in women with an annual mortality rate in the United Kingdom of over 14,000 (OPCS Monitor, 1992), and its incidence appears to be rising in the western hemisphere (Glass & Hoover, 1990). Reduction in mortality has been reported for mammographic screening (Shapiro *et al.*, 1982; Tabar *et al.*, 1985) and adjuvant chemotherapy (Early Breast Cancer Trialists' Collaborative Group, 1992*a*,*b*). However, many patients with early breast cancer will relapse with metastatic disease and about 20% of women present with locally advanced disease. There is an array of treatment options including endocrine therapy, cytotoxic chemotherapy and radiotherapy. The choice of treatment can be guided by prognostic markers, but assessment of response based on measurement of tumour size is often delayed, and consequently patients experience toxicity from ineffective treatment. $^{31}$P Magnetic Resonance Spectroscopy (MRS) may be able to monitor biochemical changes and offers the potential for early prediction of treatment response and disease relapse. It may also be useful in assessing biochemical effects of new antitumour agents that are not directly cytotoxic.

$^{31}$P MRS provides information on phospholipid metabolism, cellular energetics and intracellular pH. Studies of a wide range of human tumours in comparison with normal tissue demonstrate raised phosphomonoesters (PME) (Oberhaensli *et al.*, 1986; Cadoux-Hudson *et al.*, 1989). Serial $^{31}$P MRS studies in a patient with locally advanced breast cancer demonstrated a reduction in the level of PME in response to tamoxifen and cytotoxic chemotherapy (Glaholm *et al.*, 1989). In another report of three patients with locally advanced breast tumours, PME/ATP decreased in response to therapy (Ng *et al.*, 1989). PC and PE are intermediates in membrane phospholipid biosynthesis (Radda *et al.*, 1989) and can also be generated by growth factor signalling (Fisher *et al.*, 1991). The concentration of PME may reflect the rate of cell membrane synthesis and therefore cellular proliferation (Radda *et al.*, 1989). This is consistent with the observation of high concentration of the phosphomonoester, PE in the developing infant brain (Hope *et al.*, 1984; Younkin *et al.*, 1984) and liver (Moorcroft *et al.*, 1991), and is further supported by the increase in PME to ATP ratio during liver regeneration in the rat following 70% resection (Murphy *et al.*, 1992). However, alteration in PME associated with therapy could also be explained by a change in cellular number, particularly as in most cases there was an associated change in tumour size. The biochemical basis for therapy induced changes in PME in human breast cancer detected by $^{31}$P MRS is not clear.

The aim of this study was to assess the relationship between the level of PME measured by $^{31}$P MRS *in vivo* and proliferation in untreated, primary human breast cancer. Patients were studied the day before surgery using $^{31}$P MRS and where possible, PME composition of extracts of tumour were studied by high resolution $^{31}$P MRS to aid interpretation of the *in vivo* spectra. In addition, elderly patients treated with tamoxifen were followed with serial $^{31}$P MRS studies.

### Materials and methods

### *Patients*

Fifty-seven patients (median age 55 years, range 35–71 years) with untreated, locoregional breast cancer (UICC Stage T1-4, N0-1, M0) presenting to the Breast Unit at the John Radcliffe Hospital were studied. The study was approved by the local ethics committee, and informed consent was obtained from all subjects. Invasive carcinoma of the breast was confirmed in all cases by light microscopy of haematoxylin and eosin stained sections, and assessment of histological grading of ductal tumours was based on nuclear pleomorphism, mitotic figures and tubule formation. Maximum tumour diameter was measured from the pathological specimen. Oestrogen (ER) was determined using the dextran coated charcoal assay (Leake *et al.*, 1981) and epidermal growth factor receptor (EGFr) by the radioligand binding assay (Nicholson *et al.*, 1988). Levels greater than 10 fmol mg$^{-1}$ cytosol protein for ER, and greater than 20 fmol mg$^{-1}$ membrane protein for EGFr were taken as positive.

In addition, serial $^{31}$P MRS studies were performed in 17 elderly patients (median age 78.5 years, range 71–86 years) treated with tamoxifen 20 mg twice a day. The diagnosis of breast cancer was confirmed by fine needle aspirate cytology. $^{31}$P MRS was performed before and at intervals during tamoxifen therapy.

Correspondence: R. Kalra, Cambridge Health Authority, Addenbrooke's Hospital, Clinical Oncology Centre, Hills Road, Cambridge CB2 2QQ, UK.
Received 11 June 1992; and in revised form 10 December 1992.

*Controls*

$^{31}P$ MR spectra of normal breast were obtained from 14 volunteers (median age 44 years, range 25–82 years) with no known breast pathology using the same $^{31}P$ MR protocol as the patients.

*$^{31}P$ MRS* in vivo

Localised $^{31}P$ MR spectra of the breast were obtained on a Biospec spectrometer interfaced to a 1.9 Tesla, 60 cm clear bore superconducting magnet (Oxford Research Systems), operating at 32.7 MHz for phosphorus and 80.8 MHz for proton. The probe was a double surface coil with the 4 cm diameter receiver coil electrically isolated from and positioned 1.5 cm forward from the 10 cm diameter transmitter coil (Styles, 1988). Subjects were positioned supine and slightly rotated to one side so that the tumour was within the homogeneous region of the magnet. The magnetic field homogeneity was optimised by observing the proton signal from the region of breast to be studied. Depth resolved spectra were obtained from the tumour separate from the underlying chest wall muscle using Phase Modulated Rotating Frame Imaging (PMRFI) as originally proposed by Hoult (1979) and implemented as described by Blackledge *et al.* (1987). Briefly, a total of 16 data accumulations were acquired, the pulse sequence comprising an incremented spatial encoding pulse followed by a phase encoding pulse. Each accumulation comprised 48 transients, collected with an interpulse delay of 3 s and covering a spectral width of 2000 Hz. Using 100 watts of transmitter power, a pulse length of 180 $\mu$s for both incremental and phase encoding pulse produced an approximately 90° tip angle in the centre of the tumour. As there is an inverse relationship between spatial resolution and signal to noise ratio, the number of increments was the minimum required for adequate spatial discrimination. The phosphorus data were collected in 40 min and the study completed in less than 1 h.

The free induction decays were multiplied by an exponential line broadening of 15 Hz in the chemical shift dimension (F2). 2-D Fourier transformation was performed after zero filling and applying apodisation with a Gaussian function in the spatial dimension (F1) (Styles, 1991). From the resulting 2-D data set, tumour spectra were selected by the presence of PME (undetectable in muscle) and summed to maximise signal to noise. A frequency domain, Lorentzian line fitting routine (developed by Counsell C., MRC Radiobiology Unit, Chilton) was used to measure the area of the peaks in the $^{31}P$ MR spectrum after optimising the baseline fit using polynomial corrections. A mean of three fits was used to calculate PME relative to ATP using the ATP $\gamma$-P signal as the ATP $\beta$-P peak was distorted by off resonance effects (Blackledge & Styles, 1988). Corrections for partial saturation effects were not made. For some studies, it was necessary to correct for contamination of the tumour $\gamma$ATP signal by muscle. Assuming that PCr could not be detected in tumour, the proportion of muscle contamination was calculated from the tumour $\gamma$ATP/PCr ratio using a $\gamma$ATP/PCr ratio of 0.25 measured in chest wall skeletal muscle. The tumour PME/$\gamma$ATP ratio was then corrected as appropriate. Empirically, cases with greater than 40% muscle contamination were excluded from analysis as the large correction required was likely to introduce large errors in the value for the PME/$\gamma$ATP ratio.

*$^{31}P$ MRS* in vitro

Samples of tissue obtained from 18 tumours (including seven tumours studied by *in vivo* $^{31}P$ MRS) were frozen in liquid nitrogen within 15 min of removal at surgery until the time of extraction. The tissue was ground to a powder in liquid nitrogen and homogenised in ice cold perchloric acid (6% v/v, 4 ml $g^{-1}$ tissue). The homogenate was centrifuged and the supernatant neutralised with 3 M KOH. The resulting $KClO_4$ precipitate was removed by centrifugation. Chelex 100 (disodium salt) was added to remove the metal ions and sharpen the spectral lines, and the sample recentrifuged. The supernatant was lyophilised to dryness overnight. The resulting solid was resuspended in 3 ml of double distilled water, centrifuged to remove residual chelex 100, and adjusted to pH 8.0–8.5 as there was little variation in chemical shift in this range of pH.

Fully relaxed $^{31}P$ MR spectra were obtained on a 9.4 Tesla magnet, with a repetition time 10 s, sweep width 8064 Hz and 8K data points. Composite pulse proton decoupling was applied during acquisition (power 3 watts), and gated off during the relaxation delay to avoid distortion of spectral intensity by nuclear Overhauser effects. The number of transients accumulated to achieve good signal to noise varied with weight of tumour sample. A coaxial capillary containing methylene diphosphonate acted as a concentration standard. Chemical shifts were referred to glycerophosphocholine at 2.90 ppm (relative to PCr at 0 ppm) as this resonance is virtually unaffected by pH or ionic strength changes and was usually present in the breast tumour extracts. Peak assignments were made on the basis of well established literature on $^{31}P$ MR spectra from perchloric acid extracts (Evanochko *et al.*, 1984; Murphy, 1989) and confirmed by the addition of known compounds.

*DNA flow cytometry*

Proliferation was evaluated for all untreated breast tumours studied by $^{31}P$ MRS and normal breast tissue from reduction mammoplasty cases. Flow cytometry was performed on nuclear suspensions prepared form formalin fixed paraffin embedded sections as described previously (Camplejohn *et al.*, 1989). Fifty $\mu$m sections were dewaxed and rehydrated through a series of alcohols into double distilled water. Nuclei were extracted by the addition of pepsin (5 mg $ml^{-1}$) at 37°C for 30 min at pH 1.5. Following filtration through a 35 mm pore size nylon filter and incubation with 1 $\mu$g $ml^{-1}$ DAPI (4′6-diamidino-2-phenylindol-dihydrochloride), the samples were anlaysed using a Becton Dickinson FACS Analyser powered by a mercury arc lamp. $10^5$ particles were scanned to construct a DNA histogram. DNA index was calculated by relating DNA content of the aneuploid G0/G1 peak to that for the diploid G0/G1 peak. The SPF for diploid tumours was measured using the method of Baisch *et al.* (1975). The number of cells in S phase was calculated from a rectangle fitted between the peak channels of the G0/G1 and G2/M peaks. For the DNA aneuploid histogram, the percentage of aneuploid S phase cells as a percentage of total aneuploid cells was estimated in a similar way (Camplejohn *et al.*, 1989).

*Statistical analysis*

Non parametric tests, Mann Whitney, Kendall Rank correlation coefficient and Chi squared were used as appropriate.

### Results

*$^{31}P$ MRS* in vivo

Interpretable *in vivo* $^{31}P$ MR spectra were obtained from 31/57 (54%) breast tumours studied prior to surgery. The patient characteristics are listed in Table I. Inadequate signal to noise and/or spatial resolution prevented analysis in 26 tumours. About one third of tumours (9/26) were less than 2 cm in diameter.

Figure 1 shows a typical contour plot where the contour lines represent intensity levels of the $^{31}P$ MR signal. The tumour at the front of the spectroscopic image contains high levels of PME and PDE. There is no detectable PCr. In contrast, the muscle at the back of the image contains PCr, ATP and $P_i$. The median PME/$\gamma$ATP was 1.48 (range: 0.57–3.78) and median PDE/$\gamma$ATP was 1.65 (range: 0.44–3.89). Figure 2 shows a typical *in vivo* $^{31}P$ MR spectrum from normal breast. In all cases, there was very poor signal to

**Figure 1** PMRFI data set from breast cancer represented as a contour plot with chemical shift represented on the horizontal axis and depth along the vertical axis, and selected spectra from the tumour at the front of the plot and underlying chest wall muscle at the back of the plot. PME = phosphomonoester, Pi = inorganic phosphate, PDE = phosphodiester, ATP = adenosine triphosphate.

**Table I** Clinical details of breast cancer patients ($n = 31$)

| | |
|---|---|
| Age (years) | |
| Median | 54 |
| Range | 35-71 |
| Menopausal status | |
| Pre-menopausal | 9 |
| Post-menopausal | 22 |
| Tumour diameter | |
| ⩽2 cm | 3 |
| 2⩽5 cm | 24 |
| >5 cm | 4 |
| Histology | |
| Invasive ductal | 25 |
| Grade I | 0 |
| Grade II | 12 |
| Grade III | 12 |
| Not classified | 1 |
| Invasive lobular | 4 |
| Mixed ductal + lobular | 2 |
| Axillary node status | |
| Negative | 16 |
| Positive | 14 |
| Unknown | 1 |
| ER | |
| ⩽10 fmol mg$^{-1}$ protein | 14 |
| >10 fmol mg$^{-1}$ protein | 17 |
| EGFr | |
| ⩽20 fmol mg$^{-1}$ protein | 16 |
| >20 fmol mg$^{-1}$ protein | 13 |
| Unknown | 2 |
| DNA index | |
| Diploid | 12 |
| Aneuploid | |
| 1.1-1.9 | 15 |
| 2.0-3.0 | 4 |
| SPF median (range) | |
| $n = 29$ All | 5.3% (0.6-28%) |
| $n = 11$ Diploid | 3.8% (1.3-6.5%) |
| $n = 18$ Aneuploid | 9.4% (0.6-28%) |

noise indicating that the total level of phosphorus-containing metabolites was much lower than in breast cancer.

### $^{31}$*P MRS* in vivo *and tumour proliferation*

DNA histograms could be interpreted for 29 tumours (coefficient of variation >8% for excluded cases). Twelve (39%) were diploid and 19 aneuploid. The median (range) SPF for all tumours was 5.3% (0.6-28%). The median (range) SPF for aneuploid tumours (9.3% (0.6-28%)) was significantly higher than for diploid tumours (3.8% (1.3-6.5%)) ($P = 0.007$).

DNA histograms could be evaluated for 6/8 normal breast. All were diploid and the median (range) SPF was 1.4% (1.0-1.8%). The median SPF for normal breast was significantly lower than for both diploid ($P = 0.007$) and aneuploid tumours ($P = 0.0005$).

There was a significant association between PME/γATP and SPF ($P = 0.03$) for all tumours (Figure 3a) due to a significant relationship for the aneuploid tumours ($P = 0.01$) (Figure 3b). No relationship between SPF and PME/γATP was observed for diploid tumours (Figure 3c). Some diploid tumours with low SPF values had moderate PME/γATP ratios. No significant association was found between the relative level of PME and DNA ploidy ($P = 0.39$).

### $^{31}$*P MRS* in vivo *and other presentation features*

Median tumour diameter was 3.0 cm (range 1.5-15 cm) (Table I). Histological grade was documented for 24/25 (96%) invasive ductal carcinomas. Equal numbers were described as moderately well and poorly differentiated. Axillary lymph node metastases were present in 14/30 (45%). ER was recorded for all tumours, 17 (55%) had values greater than 10 fmol mg$^{-1}$ cytosol protein and were therefore positive. No significant association was observed between PME/γATP and

**Figure 2** $^{31}$P MR spectrum from normal breast (age 33) with very poor signal to noise.

tumour size ($P = 0.60$), histological grade ($P = 0.93$), axillary lymph node status ($P = 0.37$) or ER status ($P = 0.87$).

The median EGFr for 29 tumours was 17.2 fmol mg$^{-1}$ membrane protein (range: 0–148 fmol mg$^{-1}$ membrane protein). Sixteen (55%) were positive with values greater than 20 fmol mg$^{-1}$ membrane protein. No relationship was found between PME/γATP and the level of EGFr ($P = 0.75$) or EGFr status ($P = 0.14$).

### *$^{31}$P MRS* in vivo *and the effect of tamoxifen*

Serial $^{31}$P MRS studies were not evaluable for 14/17 patients due to poor signal to noise for seven tumours, inadequate localisation for five tumours, and refusal for repeat studies for two patients. The results for three patients with evaluable long term $^{31}$P MRS studies are presented.

*Case 1* A 82 year old woman presented with tumour measuring 6 × 6 cm in the upper part of the right breast. There was attachment to skin and no sign of fixation to pectoralis major. There was an enlarged right axillary lymph node. $^{31}$P MRS was performed the day before starting tamoxifen, at 7 days when no change in tumour size was detected, and at 42 days when progressive tumour not responding to tamoxifen was documented. Tumour size had increased to 7 × 6 cm and there were multiple skin deposits in the outer part of the breast. There was progressive increase in PME/γATP from 0.52 at day 0 to 0.80 at day 7 (Figure 4), and 1.13 at day 42. In addition, there was an improvement in signal to noise.

*Case 2* A 71 year old women presented with tumour localised to the upper outer quadrant of the right breast measuring 5 × 4 cm. Tamoxifen for 17 months stabilised tumour size. Serial $^{31}$P MRS studies detected progressive reduction in PME/γATP from 2.35 at day 0, to 2.06 at day 86, and 1.27 at day 198. The ratio of PDE/γATP fluctuated from 3.85 at day 0 to 4.90 at day 86, to 1.63 at day 198. At 17 months, the MRS study was uninterpretable due to spectral contamination from underlying chest wall muscle. Localisation was poor for this study. The calculated contribution from muscle to the tumour γATP peak was 64%.

*Case 3* A 84 year old woman presented with tumour in the upper part of the right breast measuring 5 × 7 cm. There were no palpable axillary lymph nodes. $^{31}$P MRS on day 7 detected an increase in PME/γATP (day 0: 0.29, day 7: 0.59) and PDE/γATP (day 0: 1.07; day 7: 1.47), although there had been no change in tumour size. At day 241, signal to noise in the tumour spectrum was very poor and resonances from PDE and $P_i$ could just be detected, although the tumour was 6 × 6 cm. As the quality of the shimming was similar to the previous MRS studies, the deterioration in the signal to noise was probably due to a reduction in viable tissue within the breast tumour.

### *$^{31}$P MRS* in vitro *and identification of phosphomonoester*

Figure 5 shows $^{31}$P MR spectra from a human breast cancer *in vivo* and *in vitro*. The PME region consists predominantly of phosphoethanolamine (PE) and phosphocholine (PC) with a greater contribution from PE ($n = 18$, PE/PC: median 3.02, range 1.13–5.09). There was marked variation in the concentration of PE (median: 1.07 μmol g$^{-1}$ wet wt, range: 0.23–2.91 μmol g$^{-1}$ wet wt) and PC (median: 0.29 μmol g$^{-1}$ wet

**Figure 3** **a**, PME/$\gamma$ATP *vs* S phase fraction for human breast cancer based on 29 cases. **b**, 18 aneuploid tumours; **c**, 11 diploid tumours.

wt, range: 0.11–2.21 $\mu$mol $g^{-1}$ wet wt) between tumours. This was also seen for the relative levels of PE (median: 24.6%, range: 10.75–34%) and PC (median: 9.05%, range: 2.5–27.5%) to total acid extractable phosphorus measured from the $^{31}$P MR spectra *in vitro*. The concentration of PE increased with PC ($P = 0.0001$) reflecting tumour cellularity.

Resonances from phosphorylated sugars, glycerol-3-phosphate and glycerol-6-phosphate have been observed downfield (to the left) of PE in some $^{31}$P MR spectra *in vitro*. There was some ATP hydrolysis for all tumours during the inevitable delay between surgical excision and freezing the tumour in liquid nitrogen. PME and PDE are stable at room temperature for up to 90 min (Smith *et al*., 1991).

### *$^{31}$P MRS* in vitro *and tumour proliferation*

There was no relationship between DNA ploidy and the concentration or relative levels of PE, PC or PE plus PC. For all tumour extracts no relationship was observed between SPF and the concentration of PE ($P = 0.62$), PC ($P = 0.45$) or PE plus PC ($P = 0.45$). Similarly there was no significant association between SPF and the relative levels of PE ($P = 0.49$), PC ($P = 0.34$) or PE plus PC ($P = 0.34$). This was also the case for analyses of aneuploid ($n = 11$) and diploid tumours ($n = 6$).

### Discussion

PMRFI provides depth resolved biochemical data by utilising the linear radiofrequency field produced by the transmitter coil. Calibration experiments using multicompartment phantoms show that the $^{31}$P MR signal is received from a volume of tissue approximating to a bell shape 4 cm diameter close to the coil and increasing to 8.5 cm diameter at 3 cm from the receiver coil (Dunn *et al*., 1992). The total volume is estimated to be 96 cm$^3$. For small tumours, the sensitive volume detected by the receiver coil contains both malignant and normal breast tissue. However, the paucity of $^{31}$P MR signal detected from normal breast suggests that there was negligible contribution from normal tissue to the tumour spectra.

The aneuploid tumours demonstrated a wide range of proliferation whereas the diploid tumours had low proliferative activity. The significantly greater median SPF for aneuploid tumours is in agreement with the results of thymidine labelling studies estimating SPF (McDivitt *et al*., 1985). Contamination by lymphoid and other diploid non malignant cells could lead to an underestimate for SPF for the diploid tumours. This, however, is likely to be insignificant as the median SPF for diploid tumours (3.8%) was significantly greater than for normal breast (1.4%). SPF for normal breast were in a narrow range with very low levels.

There was a strong relationship between tumour proliferation and PME/$\gamma$ATP due to a highly significant correlation for aneuploid tumours. No relationship was observed for diploid tumours. Studies have shown that abnormal DNA ploidy is indicative of aggressive growth as aneuploid tumours have a worse prognosis than diploid tumours (Hedley *et al*., 1987; Merkel & McGuire, 1990) although this finding is not universal (O'Reilly *et al*., 1990). The range of SPF values for diploid tumours (1.6–6.5%) suggests that the majority of cells in these tumours are not proliferating. The predominant cell population will determine the composition of the $^{31}$P MR spectrum. Therefore, a relationship between PME and proliferation would not be detected if the PME content of proliferating cells was not greatly elevated. In support, actively growing human breast cancer cells in culture contain approximately twice the amount of PE and PC than quiescent cells (Daly *et al*., 1987).

For the breast cancer extracts, no relationship between PE, PC or PE plus PC with SPF was observed, possibly due to the smaller number of samples (aneuploid $n = 11$, total $n = 18$).

The *in vitro* values for the phospholipid metabolites are not strictly comparable with the *in vivo* measurements but it was not possible to calculate the level of PE or PC relative to $\gamma$ATP due to ATP hydrolysis to $P_i$ during the inevitable delay between excision of tumour and storage in liquid nitrogen. Calculation of total phosphorus *in vivo* was not appropriate as the signal to noise often prevented detection of all the ATP peaks and also because the PDE peak contains resonances from membrane phospholipids.

As cellular ATP is tightly regulated (Neeman & Degani, 1989), PME relative to ATP may be indicative of intracellular levels, particularly as cellularity was highly variable as reflected by the wide range of concentrations for PE and PC. ATP production can be compromised by hypoxia and nutrient deficiency (Freyer *et al*., 1990), but for human tumours this represents a small proportion of the whole tumour (Sutherland *et al*., 1988) and would not significantly affect the tumour spectrum.

The increase in PME/$\gamma$ATP for the tumour resistant to tamoxifen is compatible with an association between PME/$\gamma$ATP and proliferation. Tamoxifen was associated with stable disease for the other two cases. The progressive reduction of PME/$\gamma$ATP for the second case is consistent with the cytostatic action of tamoxifen. Replacement of viable cells by fibrosis could explain marked reduction of intensity of $^{31}$P MR signal detected by $^{31}$P MRS for the third case. The initial increase in PME/$\gamma$ATP may represent an initial tumour flare due to the weak oestrogenic activity of tamoxifen.

The PME signal has contributions principally from PE and PC with PE predominating. Other human tumours such as meningioma (Kalra *et al*., 1991) and hepatic lymphoma (Dixon *et al*., 1991) are also rich in PE. For regenerating rat

**Figure 4** $^{31}$P MR spectra from Case 1 studied **a**, before starting tamoxifen and **b**, 7 days later.

liver, the increase in the ratio of PE/PC from 12 h to 48 h following partial hepatectomy appears to be mediated by growth factor signalling as there was a parallel increase in the second messenger, diacylglycerol (Murphy *et al.*, 1992). Both PE and PC can be produced by hydrolysis of the membrane phospholipids, phosphatidylethanolamine (Kiss & Anderson, 1989) and phosphatidylcholine (Pelech & Vance, 1989) respectively, by phospholipase C. Hydrolysis of phosphatidylcholine by phospholipase D produces choline and phosphatidic acid which is then cleaved to diacylglycerol. It is not clear if the phospholipase C and phospholipase D pathways are activated together or preferentially (Price *et al.*, 1989). In human dermal fibroblasts the production of diacylglycerol by EGF occurs primarily by phospholipase C (Fisher *et al.*, 1991). However, the source of PME in breast tumours is unlikely to be due to cell signalling, as there was no relationship between the pool of PME and EGFr level or status. The role of phospholipid metabolites in cellular signalling is not

**Figure 5** **a**, *In vivo* $^{31}$P MR spectra from a patient with breast cancer. **b**, High resolution $^{31}$P MR spectrum of a perchloric acid extract of a sample of breast cancer from the same patient. PE = phosphoethanolamine, PC = phosphocholine, GPE = glycerophosphoethanolamine, GPC = glycerophosphocholine.

excluded as the change in pool size is likely to be small, or absent as the fluxes of metabolites in and out of the pool could be in balance.

The presence of high levels of PME in tumours may reflect their role as membrane phospholipid precursors. It has been suggested that the increased demand for membrane synthesis in rapidly dividing cells leads to an increase in the synthetic precursors (Radda *et al*., 1989). The CDP-choline pathway is the predominant route for phosphatidylcholine synthesis in eucaryotic cells (Figure 6). Upregulation of choline kinase with increased production of PC (Warden & Friedken, 1985), the precursor for the rate limiting step in this pathway may account for increased PC in rapidly growing cells. In contrast, the contribution of the CDP-ethanolamine pathway to phosphatidylethanolamine synthesis appears to depend upon the supply of ethanolamine, at physiological levels this route contributes about 30% of synthesis (Miller & Kent, 1986). The CDP-choline and ethanolamine pathways have distinct

**Figure 6** Pathways for biosynthesis of the major membrane phospholipids in mammalian cells.

enzymes (Pelech & Vance, 1984) and appear to be under separate control as the incorporation of $^{14}$C-ethanolamine into phospholipid is not directly related to the activity of the enzymes involved (Groener *et al.*, 1979). Decarboxylation of phosphatidylserine with the production of ethanolamine is known to be an important source of phosphatidylethanolamine (Bishop & Bell, 1988). Ethanolamine is also produced by base exchange of free serine with the polar head group of phosphatidylethanolamine, the major route for synthesis of phosphatidylserine. High concentrations of ethanolamine are toxic in cellular systems (Kaiho & Mizuno, 1985) and could therefore be disposed of by conversion to PE in the CDP-ethanolamine pathway.

In summary, this study demonstrates a significant association between the relative level of PME to γATP and proliferation for aneuploid tumours. The PME region contains predominantly PE with a smaller contribution from PC. The presence of these compounds may be a consequence of a shift in the balance of phospholipid metabolism to synthesis with more rapid synthesis in the faster growing tumours. Changes in PME/γATP observed following tamoxifen may be consistent with the cytostatic effects of this drug. More cases need to be studied but the low success rate here reflects the small numbers of cases in published accounts. $^{31}$P MRS may only be applicable for monitoring therapy in large tumours, assessing novel agents and their metabolic effects.

Alteration in membrane composition can occur with malignancy (Bergelson *et al.*, 1970, 1974) and could influence the levels of phospholipid precursors. We are therefore studying the relationship between PME and membrane phospholipids.

This work was funded by the Medical Research Council, Imperial Cancer Research Fund and by the John Radcliffe Hospital.

### References

BAISCH, H., GOHDE, W. & LINDEN, W.A. (1975). Analysis of PCP-data to determine the fraction of cells in the various phases of the cell cycle. *Radiat. Environ. Biophys.*, **12**, 31–39.

BERGELSON, L.D., DYATLOVITSKAYA, E.V., TORKHOVSKAYA, T.I., SOROKINA, I.B. & GORKOVA, N.P. (1970). Phospholipid composition of membranes in the tumour cells. *Biochim. Biophys. Acta*, **210**, 287–298.

BERGELSON, L.D., DYATLOVITSKAYA, E.V., SOROKINA, I.B. & GORKOVA, N.P. (1974). Phospholipid composition of mitochondria and microsomes from regenerating rat liver and hepatoma of different growth rate. *Biochim. Biophys. Acta*, **360**, 361–365.

BISHOP, W.R. & BELL, R.M. (1988). Assembly of phospholipids into cellular membranes: biosynthesis, transmembrane movement and intracellular location. *Ann. Rev. Cell Biol.*, **4**, 579–610.

BLACKLEDGE, M.J., RAJAGOPALAN, B., OBERHAENSLI, R.D., BOLAS, N.M., STYLES, P. & RADDA, G.K. (1987). Quantitative studies of human cardiac metabolism by $^{31}$P rotating frame NMR. *Proc. Natl Acad. Sci. USA*, **84**, 4283–4287.

BLACKLEDGE, M.J. & STYLES, P. (1988). Effect of pulse angle imperfection and resonance offset in the PMRFI experiment. *J. Magn. Reson.*, **77**, 203–222.

CADOUX-HUDSON, T.A.D., BLACKLEDGE, M.J., RAJAOPLAN, B., TAYLOR, D.J. & RADDA, G.K. (1989). Human primary brain tumour metabolism *in vivo*: a phosphorus magnetic resonance spectroscopy study. *Br. J. Cancer*, **60**, 430–436.

CAMPLEJOHN, R.S., MACCARTNEY, J.C. & MORRIS, R.W. (1989). Measurement of S-phase fractions in lymphoid tissue comparing fresh versus paraffin-embedded tissue and 4',6'-diamidino-2 phenolindole dihydrochloride versus propidium lodide staining. *Cytometry*, **10**, 410–416.

DALY, P.F., LYON, F.C., FAUSTINO, P.J. & COHEN, J.S. (1987). Phospholipid metabolism in cancer cells monitored by $^{31}$P NMR spectroscopy. *J. Biol. Chem.*, **262**, 14875–14878.

DIXON, R., ANGUS, P.W., RAJAGOPALAN, B. & RADDA, G.K. (1991). Abnormal phosphomonoester signals in $^{31}$P NMR spectra from patients with hepatic lymphoma. A possible marker of liver infiltration and response to therapy. *Br. J. Cancer*, **63**, 953–958.

DUNN, J.F., KEMP, G.J. & RADDA, G.K. (1992). Depth selective quantification of phosphorus metabolites in human calf muscle. *NMR in Biomed.*, **5**, 154–160.

EARLY BREAST CANCER TRIALISTS' COLLABORATIVE GROUP (1992*a*). Systemic treatment of early breast cancer by hormonal, cytotoxic, or immune therapy. *Lancet*, **339**, 1–15.

EARLY BREAST CANCER TRIALISTS' COLLABORATIVE GROUP (1992*b*). Systemic treatment of early breast cancer by hormonal, cytotoxic or immune therapy. *Lancet*, **339**, 71–85.

EVANOCHKO, W.T., SAKAI, T.T., NG, T.T., KRISHNA, N.R., KIM, H.D., ZEIDLER, R.B., GHANTA, V.K., BROCKMAN, R.W., SCHIFFER, L.M., BRAUNSCHWEIGER, P.G. & GLICKSON, J.D. (1984). NMR study of *in vivo* RIF-1 tumours. Analysis of perchloric extracts and identification of $^{1}$H, $^{31}$P and $^{13}$C resonances. *Biochim. Biophys. Acta*, **805**, 104–116.

FISHER, G.J., HENDERSON, P.A., VOORHEES, J.J. & BALDASSARE, J.J. (1991). Epidermal growth factor-induced hydrolysis of phosphatidylcholine by phospholipase D and phospholipase C in human dermal fibroblasts. *J. Cell. Physiol.*, **146**, 309–317.

FREYER, J., FINK, N., NEEMAN, M. & SILLERUD, L. (1990). Phosphorus NMR spectroscopy of multicellular tumour spheroids: a model for interpreting *in vivo* tumour spectra. *Proceedings 9th Meeting of the Soc. Magn. Reson. Med., New York*, p. 855.

GLAHOLM, J., LEACH, M.O., COLLINS, D.J., MANSI, J., SHARP, J.C., MADDEN, A., SMITH, I.E. & MCCREADY, V.R. (1989). *In vivo* $^{31}$P magnetic resonance spectroscopy for monitoring treatment response in breast cancer. *Lancet*, **i**, 1326–1327.

GLASS, A.G. & HOOVER, R.N. (1990). Rising incidence of breast cancer: relationship to stage and receptor status. *J. Natl Cancer Inst.*, **82**, 693–696.

GROENER, J.E.M., KLEIN, W. & VAN GOLDE, L.M.G. (1979). The effect of fasting and refeeding on the composition and synthesis of triacylglycerols, phosphatidylcholines and phosphatidylethanolamines in rat liver. *Arch. Biochem. Biophys.*, **198**, 287–295.

HEDLEY, D.W., RUGG, C.A. & GELBER, R.D. (1987). Association of DNA index and S phase fraction with prognosis of node positive early breast cancer. *Cancer Res.*, **47**, 4729–4735.

HOPE, P.L., CADY, E.B., TOFTS, P.S., HAMILTON, P.A. & COSTELLO, A.M. (1984). Cerebral energy metabolism studied with phosphorus NMR spectroscopy in normal and birth asphyxiated infants. *Lancet*, **2**, 366–370.

HOULT, D.I. (1979). Rotating frame zeugmatography. *J. Magn. Reson.*, **33**, 183–197.

KAIHO, S. & MIZUNO, K. (1985). Inhibition of Friend cell erythroid differentiation by modification of membrane phospholipid composition by choline analogues. *Biochim. Biophys. Acta*, **838**, 175–178.

KALRA, R., WADE, K.E., CADOUX-HUDSON, T.A.D., PARKES, H.G., MCDONALD, B., ESIRI, M. & RADDA, G.K. (1991). NMR study of phospholipid metabolism of human meningioma. *Proceedings 10th Meeting of the Soc. Magn. Reson. Med., San Francisco*, p. 628.

KISS, Z. & ANDERSON, W.B. (1989). Phorbol ester stimulates the hydrolysis of phosphatidylethanolamine in Leukaemic HL-60, NIH 3T3 and baby hamster kidney cells. *J. Biol. Chem.*, **264**, 1483–1487.

LEAKE, R.E., LAING, L., CALMAN, K.C., MACBETH, F.R., CRAWFORD, D. & SMITH, D.C. (1981). Oestrogen receptor status and endocrine therapy of breast cancers: response rates and status stability. *Br. J. Cancer*, **43**, 59–66.

MCDIVITT, R.W., STONE, K.R., BRUCE, C.R. & MEYERS, J.S. (1985). A comparision of human breast cancer cell kinetics measured by flow cytometry and thymidine labelling. *Lab. Invest.*, **52**, 287–291.

MERKEL, D.E. & MCGUIRE, W.L. (1990). Ploidy, proliferative activity and prognosis. DNA flow cytometry of solid tumours. *Cancer*, **65**, 1194–1205.

MILLER, M.A. & KENT, C. (1986). Characterisation of the pathways for phosphatidylethanolamine biosynthesis in Chinese Hamster ovary mutant and parental cell lines. *J. Biol. Chem.*, **261**, 9753–9761.

MOORCRAFT, J., BOLAS, N.M., IVES, N.K., OUWERKERK, R., SMYTH, J., RAJAGOPALAN, B., HOPE, P.L. & RADDA, G.K. (1991). Global and depth resolved phosphorus magnetic resonance spectroscopy to predict outcome after birth asphyxia. *Arch. Dis. Childhood*, **66**, 1119–1123.

MURPHY, E.J. (1989). D.Phil. Thesis. University of Oxford.

MURPHY, E.J., BRINDLE, K.M., RORISON, C.J., DIXON, R.M., RAJAGOPALAN, B. & RADDA, G.K. (1992). Changes in phosphatidylethanolamine metabolism in regenerating rat liver as measured by $^{31}$P-NMR. *Biochim. Biophys. Acta*, **1135**, 527–534.

NEEMAN, M. & DEGANI, H. (1989). Early estrogen-induced metabolic changes and their inhibition by actinomycin D and cycloheximide in human breast cancer cells: $^{31}$P and $^{13}$C studies. *Proc. Natl. Acad. Sci. USA*, **86**, 5585–5589.

NG, T.C., MAJORS, A.W., VIJAYKUMAR, S., BALDWIN, N.J., MAJORS, A.W., KARALIS, I., MEANEY, T.F., SHIN, K.H., THOMAS, F.J. & TUBBS, R. (1989). Therapeutic response of breast carcinoma monitored by $^{31}$P MRS *in situ*. *Magn. Reson. Med.*, **10**, 125–134.

NICHOLSON, S., SAINSBURY, J.R.C., NEEDHAM, G.K., CHAMBERS, P., FARNDON, J.R. & HARRIS, A.L. (1988). Quantitative assays of epidermal growth factor receptor in human breast cancer: cut-off points of clinical relevance. *Int. J. Cancer*, **42**, 36–41.

OBERHAENSLI, R.D., BORE, P.J., RAMPLING, R.P., HILTON-JONES, D., HANDS, L.J. & RADDA, G.K. (1986). Biochemical investigation of human tumours *in vivo* with phosphorus-31 magnetic resonance spectroscopy. *Lancet*, **i**, 8–11.

OFFICE OF POPULATION CENSUSES AND SURVEYS MONITOR. (1992). Series DH2 92/1. London, HMSO.

O'REILLY, S.M., CAMPLEJOHN, R.S., BARNES, D.M., MILLIS, R.R., ALLEN, D., RUBENS, R.D. & RICHARDS, M.A. (1990). DNA index, S-phase fraction, histological grade and prognosis in breast cancer. *Br. J. Cancer*, **61**, 671–674.

PELECH, S.L. & VANCE, D.E. (1984). Regulation of phosphatidylcholine biosynthesis. *Biochim. Biophys. Acta*, **779**, 217–251.

PELECH, S.L. & VANCE, D.E. (1989). Signal transduction via phosphatidylcholine cycles. *Trends in Biochem. Sci.*, **14**, 28–30.

PRICE, B.D., MORRIS, J.D.H. & HALL, A. (1989). Stimulation of phosphatidylcholine breakdown and diacylglycerol production by growth factors in Swiss 3T3 cells. *Biochem. J.*, **264**, 509–515.

RADDA, G.K., RAJAGOPALAN, B. & TAYLOR, D.J. (1989). Biochemistry *in vivo*: an appraisal of clinical magnetic resonance spectroscopy. *Mag. Res. Q.*, **5**, 122–151.

SHAPIRO, S., VENET, W., STRAX, L., VENET, L. & ROESER, R. (1982). Ten to fourteen year effect of screening on breast cancer mortality. *J. Natl Cancer Inst.*, **69**, 349–355.

SMITH, T.A.D., GLAHOLM, J., LEACH, M.O., MACHIN, L. & MCCREADY, V.R. (1991). The effect of intra-tumour heterogeneity on the distribution of phosphorus-containing metabolites within human breast tumours: an *in vitro* study using $^{31}$P NMR spectroscopy. *NMR in Biomed.*, **4**, 262–267.

STYLES, P. (1988). Passive electrical isolation of double coil probes for localised spectroscopy and imaging. *NMR in Biomed.*, **1**, 61–66.

STYLES, P. (1991). Quantitative spectroscopy using multiple surface coil probes. *Magn. Reson. Med.*, **17**, 3–9.

SUTHERLAND, R.M., RASEY, J.S. & HILL, R.P. (1989). Tumour biology. *Am. J. Clin. Oncol.*, **11**, 253–274.

TABAR, L., FAGERBERG, C.J.C., GAD, A., BALDETROP, L., HOLMBERG, L.H., GRONTOFT, O., LJUNGQUIST, U., LUNDSTROM, B., MANSON, J.C., EKLUND, G., DAY, N.E. & PETTERSSON, F. (1985). Reduction in mortality from breast cancer after mass screening with mammography. Randomised trial from the Breast Cancer Screening Working Group of the Swedish National Board of Health and Welfare. *Lancet*, **i**, 829–832.

WARDEN, C.H. & FRIEDKEN, M. (1985). Regulation of choline kinase activity and phosphatidylcholine biosynthesis by mitogenic growth factors ion 3T3 fibroblasts. *J. Biol. Chem.*, **260**, 6006–6011.

YOUNKIN, D.P., DELUORAI-PAPADOPOULUS, M. & LEONARD, J. (1984). Unique aspects of human newborn metabolism evaluated with phosphorus nuclear magnetic resonance spectroscopy. *Ann. Neurol.*, **16**, 581–586.